# Pathogenic tau does not drive activation of the unfolded protein response

**DOI:** 10.1074/jbc.RA119.008263

**Published:** 2019-05-03

**Authors:** Aleksandra P. Pitera, Ayodeji A. Asuni, Vincent O'Connor, Katrin Deinhardt

**Affiliations:** From ‡ Biological Sciences, University of Southampton, Southampton SO17 1BJ, United Kingdom and; § Systems Biology - Symptoms, H. Lundbeck A/S, 2500 Valby, Denmark

**Keywords:** neuron, Tau protein, tauopathy, transgenic mice, unfolded protein response (UPR), neurodegenerative disease, protein misfolding, frontotemporal dementia, primary hippocampal neuron

## Abstract

The unfolded protein response (UPR) is commonly associated with a range of neurodegenerative diseases, and targeting UPR components has been suggested as a therapeutic strategy. The UPR surveys protein folding within the endoplasmic reticulum. However, many of the misfolded proteins that accumulate in neurodegeneration are localized so that they do not directly cause endoplasmic reticulum triggers that activate this pathway. Here, using a transgenic mouse model and primary cell cultures along with quantitative PCR, immunoblotting, and immunohistochemistry, we tested whether the UPR is induced in *in vivo* and *in vitro* murine models of tauopathy that are based on expression of mutant tau^P301L^. We found no evidence for the UPR in the rTg4510 mouse model, in which mutant tau is transgenically expressed under the control of tetracycline-controlled transactivator protein. This observation was supported by results from acute experiments in which neuronal cultures expressed mutant tau and accumulated misfolded cytoplasmic tau aggregates but exhibited no UPR activation. These results suggest that the UPR is not induced as a response to tau misfolding and aggregation despite clear evidence for progressive cellular dysfunction and degeneration. We propose that caution is needed when evaluating the implied significance of the UPR as a critical determinant across major neurodegenerative diseases.

## Introduction

One of the common features of many neurodegenerative diseases is the presence of misfolded proteins that aggregate and disturb cellular homeostasis (1, 2). To prevent the consequences of misfolded protein aggregation, cells activate protective mechanisms such as the unfolded protein response (UPR)^2^ (2); however, chronic activation of the UPR is suggested to lead directly to pathology (2). The UPR is induced upon the appearance of unfolded/misfolded proteins within the endoplasmic reticulum (ER). Its activation has been reported in Alzheimer's disease (AD), Parkinson's disease, and ALS (3–6). However, in these diseases, the misfolded aggregates are mainly cytoplasmic or extracellular. Thus, the misfolding pathway is independent of the ER lumen, where the key triggers for the UPR are localized (6). Although it remains a possibility that indirect activation of the ER-based UPR may occur (7), it is unclear how the disturbed cytoplasmic proteostasis associated with neurodegeneration couples to an UPR.

There are three branches of the UPR: inositol-requiring enzyme-1 (IRE1), protein kinase R–like endoplasmic reticulum kinase (PERK), and activating transcription factor 6 (ATF6); together, they increase the protein folding capacity of the ER, decrease global protein synthesis, and stimulate ER-associated degradation (8). Activation of all three cascades leads to increased expression of the ER-resident chaperone BiP and to other events, including splicing of the transcription factor XBP-1 and phosphorylation of the translation factor eIF2α, which leads to inhibition of mRNA translation (1). All of these measures aim to alleviate misfolding-induced stress, and when they fail, it can result in dysfunction and, eventually, death of the cell (2).

A well-characterized model of neurodegeneration associated with the accumulation of dysfunctional protein is the rTg4510 mouse model of tauopathy, in which mice express tau^P301L^, one of the most common human tau mutations underpinning frontotemporal dementia (9). As they age, rTg4510 mice show behavioral changes, cytosolic accumulation of neurofibrillary tangles, and atrophy of the forebrain (10, 11). The well-characterized progressive nature of the pathology enables sampling of tissues from distinct phases of the disease, allowing molecular investigation of the accumulating dysfunction.

In this study, we used rTg4510 mice to investigate the mechanisms underlying UPR activation in response to cytosolic protein aggregation but found no evidence for tau-triggered UPR activation. rTg4510 mice are generated by crossing two lines of transgenic mice: one expressing the tetracycline-controlled transactivator protein (tTA) driven by the calcium/calmodulin-dependent protein kinase II promoter and the second expressing the tetracycline-responsive promoter element placed upstream of the cDNA encoding human tau^P301L^ (10). To distinguish the effects exerted by mutant tau from those that were driven by expression of the transactivator protein, we compared animals overexpressing human tau not only with WT mice but also with mice carrying the tTA element.

To probe tau^P301L^-dependent UPR induction in a more acute model with no confound resulting from transactivator expression, we investigated primary hippocampal neurons expressing human tau^P301L^, which misfolds when expressed in primary neurons, or tau^WT^. We found that, although primary hippocampal neurons are capable of inducing the UPR, they fail to do so in response to tau misfolding. Thus, our data indicate that pathogenic tau fails to mount an evidenced UPR in distinct models of chronic and acute tau-mediated dysfunction.

## Results

### rTg4510 mice are characterized by progressive neurodegeneration

**Figure 1.**
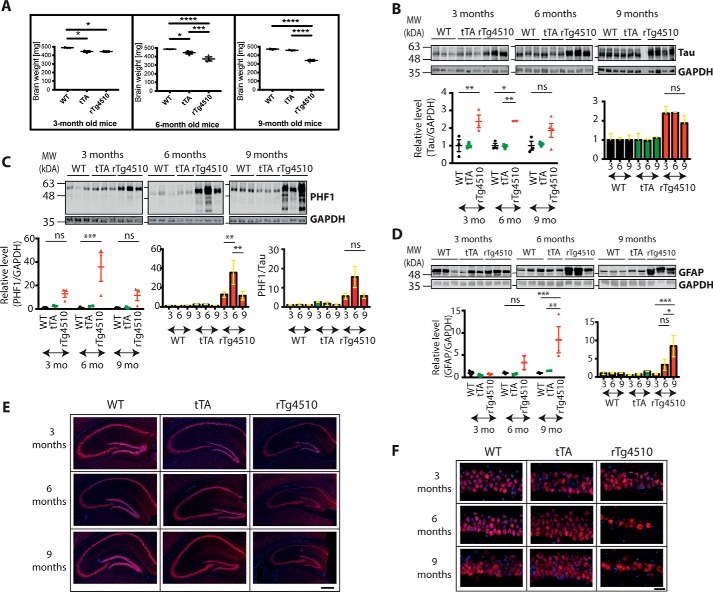
**rTg4510 mice can be characterized by progressive pathology.** *A*, brain weight changes for 3-, 6-, and 9-month-old WT, tTA, and rTg4510 mice. *Error bars* are S.E. *, *p* < 0.05; ***, *p* < 0.001; ****, *p* < 0.0001. *n* = 2–4. *B*, Western blotting showing the total level of tau protein in 3-, 6-, and 9-month-old WT, tTA, and rTg4510 mice. The blots were reprobed with GAPDH antibody to illustrate equivalent protein loading. Each lane corresponds to a separate animal. *Error bars* are S.E. *, *p* < 0.05; **, *p* < 0.01; *ns*, not significant. *MW*, molecular weight; *mo*, months. *C*, Western blot showing the level of phosphorylated tau in 3-, 6-, and 9-month-old WT, tTA, and rTg4510 mice. A PHF1 antibody directed against Ser-396/Ser-404 was used. The blots were reprobed with GAPDH antibody to illustrate equivalent protein loading. The level of p-tau was quantified against GAPDH (*left* and *center panels*) and against total tau (*right panel*). Each lane corresponds to a separate animal. *Error bars* are S.E. **, *p* < 0.01; ***, *p* < 0.001. *D*, Western blot showing the level of GFAP in 3-, 6-, and 9-month-old WT, tTA, and rTg4510 mice. The blots were reprobed with GAPDH antibody to illustrate equivalent protein loading. Each lane corresponds to a separate animal. *Error bars* are S.E. *, *p* < 0.05; **, *p* < 0.01; ***, *p* < 0.001. *E*, immunohistochemical staining of 3-, 6-, and 9-month-old WT, tTA, and rTg4510 mice. NeuN antibody was used to stain the neuronal nuclei (*red*), and Hoechst stain was used to stain the nuclei of all cell types (*blue*). *Scale bar* = 500 μm. *F*, high-resolution images of the CA1 region of 3-, 6-, and 9-month-old WT, tTA, and rTg4510 mice stained with NeuN antibody. *Scale bar* = 20 μm.

We benchmarked the tissue utilized to investigate UPR activation against the previously characterized disease progression in rTg4510 mice (10, 11). The weight of the brains used in this study was decreased in 6- and 9-month-old mice compared with age-matched WT mice, with two-way ANOVA revealing an effect of age (F_(2, 19)_ = 8.296, *p* = 0.0026), genotype (F_(2, 19)_ = 59.7, *p* < 0.0001), and age-genotype interaction (F_(4, 19)_ = 8.172, *p* = 0.0005) on brain weight (Fig. 1*A*). The brain weight was also decreased in 3-month-old tTA mice compared with the age-matched WT (*p* = 0.0319). However, there was no further decrease in brain weight in tTA mice over time, whereas rTg4510 mice presented a progressive decrease. This is consistent with an observation made in a previous study (12) and highlights both a tTA-dependent effect and an additional and more protracted tau-mediated pathology. This alerted us to potential stress-related tTA effects independent of the tau dysfunction. To control for this, our study compared WT, tTA, and tTA::tau^P301L^ transgenic cohorts.

We assessed the tau load in these cohorts by measuring the level of total and phosphorylated tau (Ser-396/404) (Fig. 1, *B* and *C*). In accordance with previously published data (10), an increased level of tau was observed in rTg4510 mice, whereas the expression and phosphorylation levels in tTA and WT mice were comparable. Statistical analysis showed a genotype effect on the level of total tau (F_(2, 18)_ = 19.17, *p* < 0.0001) and phospho-tau (F_(2, 17)_ = 16.1, *p* = 0.0001). A slight decrease in total tau and a significant decrease in phospho-tau was observed between 6- and 9-month-old rTg4510 mice (*p* = 0.0044). Therefore, we quantified the p-tau levels not only relative to GAPDH but also relative to total tau. In both instances, a decreased level of p-tau was observed at 9 months compared with the level at 6 months. This observation had been made previously and had been ascribed to the progressive loss of neurons in rTg4510 mice and, in particular, the loss of neurons bearing a high tangle load (10).

The level of GFAP was also determined to investigate the appearance of astrogliosis. The expression of this astrocytic marker was comparable between all genotypes examined in 3-month-old mice. The level of GFAP was higher in 6-month-old rTg4510 mice, and it was further increased at the 9-month time point (Fig. 1*D*). Two-way ANOVA revealed an effect of age (F_(2, 15)_ = 4.62, *p* = 0.0273) and genotype (F_(2, 15)_ = 7.089, *p* = 0.0068) and also age–genotype interaction (F_(4, 15)_ = 3.4, *p* = 0.0361) on the GFAP level. The increased GFAP level is a direct indication of increasing pathology and has been reported by others (13).

The transgene disrupts a range of forebrain structures, and here we examined the hippocampus, reported to be one of the most affected regions in the rTg4510 model (10). The brain slices were stained with the neuronal marker, NeuN (Fig. 1*E*, *F*). The staining revealed progressive atrophy of the whole hippocampal formation, with the CA1 region being the most affected, confirming data published previously (10, 12). Thinning of the dentate gyrus of tTA mice was also observed, which is in accordance with published observations (12).

Overall, these results confirm that our rTg4510 tissue displays the established features of progressive neurodegeneration accompanied by an increased load of phosphorylated tau and astrogliosis. Furthermore, they reveal the presence of tTA-dependent signaling, which highlights the need for an additional control when probing tissue from rTg4510 mice for previously uninvestigated parameters.

### The IRE1 and PERK branches are not activated in rTg4510 mice

**Figure 2.**
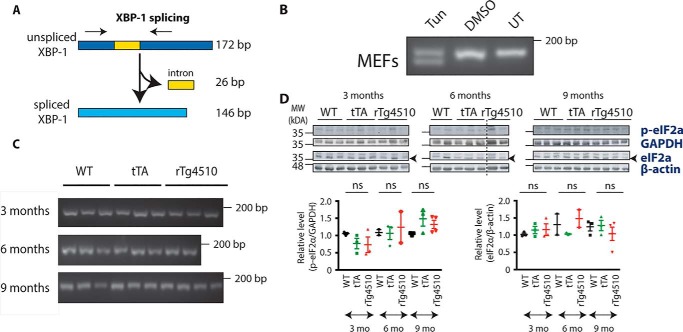
**Neither IRE1 nor the PERK pathway is activated in rTg4510 animals.** *A*, schematic illustrating splicing of the XBP-1 gene, leading to formation of a shorter spliced form and a 26-bp-long intron. The *black arrows* indicate where the primers bind so that they can amplify the unspliced and spliced form. *B*, cDNA from MEFs treated with tunicamycin (*Tun*), DMSO (vehicle treatment), or left untreated (*UT*) was subjected to PCR with XBP-1 primers detecting both unspliced and spliced forms of XBP-1. The products were resolved on a 2.5% agarose gel. *C*, cDNA from 3-, 6-, and 9-month-old WT, tTA, and rTg4510 mice was subjected to PCR with XBP-1 primers detecting both unspliced and spliced forms of XBP-1. The products were resolved on a 2.5% agarose gel. *n* = 2–4. *D*, Western blot showing the level of p-eIF2α and total eIF2α protein in the cortex of 3-, 6-, and 9-month-old WT, tTA, and rTg4510 mice. The blots were reprobed with GAPDH or actin antibody to illustrate equivalent protein loading. Each lane corresponds to a separate animal. The *dashed line* indicates splicing of the scan. *Error bars* are S.E. *MW*, molecular weight; *mo*, months; *ns*, not significant.

Next, we proceeded to investigate whether the IRE1 branch of the UPR is induced in the hippocampus of rTg4510 mice. When activated, IRE1 leads to alternative splicing of XBP-1 mRNA (Fig. 2*A*), and the spliced form activates transcription of genes that play a role in ER quality control (14). To verify whether the splicing event takes place in rTg4510 mice, primers detecting both spliced and unspliced forms were designed, and their specificity was confirmed using tunicamycin-treated mouse embryonic fibroblasts (MEFs) (Fig. 2*B*). Resolving the PCR products on agarose gel revealed the presence of two bands, with the upper one corresponding to the unspliced form and the lower band, devoid of the 26-bp intron, corresponding to the spliced variant of XBP-1. Both spliced and unspliced species were present in the tunicamycin-treated sample, with only one XBP-1 band, corresponding to the unspliced form, present in vehicle-treated and untreated MEFs. This result confirms the ability of the primers to detect XBP-1 splicing. The primers were then used to verify whether splicing occurs in the hippocampus of rTg4510 mice. We did not observe the band corresponding to the spliced form of XBP-1 at any of the time points investigated (Fig. 2*C*).

To further investigate UPR activation in rTg4510 mice, we decided to determine the level of phosphorylated eukaryotic initiation factor 2α (eIF2α). Upon UPR activation, PERK phosphorylates eIF2α, which results in inhibition of mRNA translation and alleviation of ER stress (15). The Western blotting results showed no detectable p-PERK (Fig. S1) and no increase in p-eIF2α in rTg4510 mice (Fig. 2*D*). Two-way ANOVA revealed the age effect (F_(2, 17)_ = 5.016, *p* = 0.0194) but failed to find a genotype effect (F_(2, 17)_ = 0.04761, *p* = 0.9536) or an age–genotype interaction (F_(4, 17)_ = 1.457, *p* = 0.2588). Together, this suggests that the IRE1 and PERK branches of the UPR are not activated in rTg4510 brains.

### The expression level of shared UPR markers is not increased in rTg4510 mice

**Figure 3.**
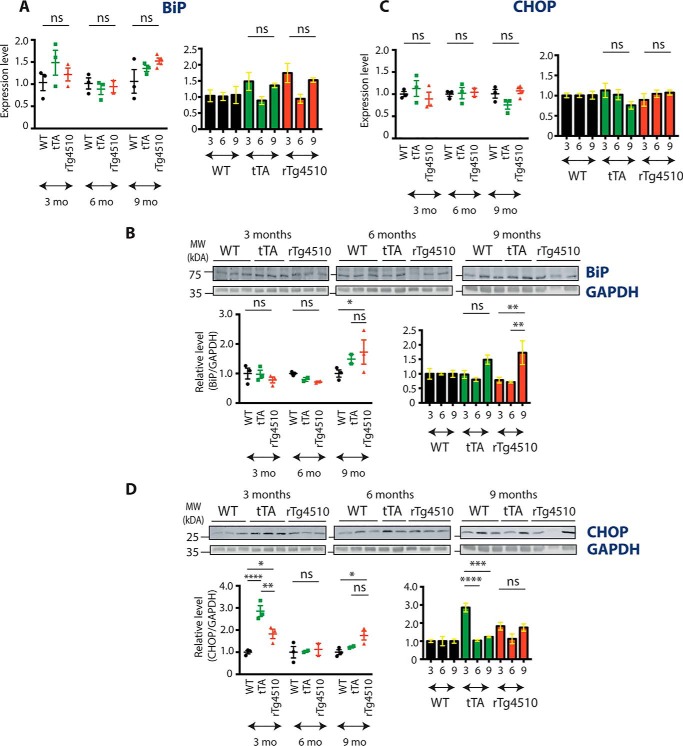
**BiP and CHOP expression in rTg4510 animals.** *A*, qPCR results showing the expression level of the BiP transcript in 3-, 6-, and 9-month-old WT, tTA, and rTg4510 animals. *Error bars* are S.E. *n* = 2–4. *mo*, months; *ns*, not significant. *B*, Western blot showing the level of BiP protein in 3-, 6-, and 9-month-old WT, tTA, and rTg4510 mice. The blots from Fig. 1*D* were reprobed with BiP antibodies; the GAPDH signal (see also Fig. 1*D*) illustrates equivalent protein loading. Each lane corresponds to a separate animal. *Error bars* are S.E. *, *p* < 0.05; **, *p* < 0.01. *MW*, molecular weight. *C*, qPCR results showing the expression level of the CHOP transcript in 3-, 6-, and 9-month-old WT, tTA, and rTg4510 animals. *Error bars* are S.E. *n* = 2–4. *D*, Western blot showing the level of CHOP protein in 3-, 6-, and 9-month-old WT, tTA, and rTg4510 mice. The blots from Figs. 1*D* and 3*B* were reprobed with CHOP antibodies; GAPDH (see also Figs. 1*D* and 3*B*) illustrates equivalent protein loading. Each lane corresponds to a separate animal. *Error bars* are S.E. *, *p* < 0.05; **, *p* < 0.01; ****, *p* < 0.0001.

To further examine the UPR in rTg4510 mice, we focused on BiP, an ER chaperone that is robustly transcribed upon UPR activation and that is involved in all three arms of the response. qPCR and Western blot were used to determine mRNA and protein levels, respectively. The mRNA level was not different between rTg4510, WT, and tTA mice (Fig. 3*A*). Two-way ANOVA revealed an age effect (F_(2, 18)_ = 3.656, *p* = 0.0465) but no effect of genotype (F_(2, 18)_ = 1.357, *p* = 0.2825) and no interaction (F_(4, 18)_ = 1.266, *p* = 0.3196) between age and genotype on the BiP mRNA level. Protein expression was significantly increased in 9-month-old rTg4510 mice compared with 9-month-old WT mice (*p* = 0.0351) (Fig. 3*B*). However, there was no difference in BiP protein level between tTA and rTg4510 mice (*p* = 0.7004), suggesting that the increased BiP expression is not due to the presence of mutant human tau.

Finally, we focused on CHOP, which is involved in UPR-induced cell death. Expression of CHOP can be induced by all three UPR branches (16) as well as by other stressors, including mitochondrial and oxidative stress (16). Similar to BiP, its mRNA and protein expression levels were determined using qPCR and Western blotting, respectively. No changes at the transcript level were found at any of the time points investigated (Fig. 3*C*). Two-way ANOVA failed to find an age effect (F_(2, 18)_ = 0.4137, *p* = 0.6673), genotype effect (F_(2, 18)_ = 0.1025, *p* = 0.9031), or an age–genotype interaction (F_(4, 18)_ = 1.832, *p* = 0.1660). In contrast, statistical analysis of CHOP protein revealed the effect of both age (F_(2, 15)_ = 14.31, *p* = 0.0003) and genotype (F_(2, 15)_ = 11.21, *p* = 0.0011) as well as an age–genotype interaction (F_(4, 15)_ = 7.911, *p* = 0.0012) (Fig. 3*D*). Tukey's post hoc test further showed that the CHOP level was increased in 9-month-old rTg4510 mice compared with the WT (*p* = 0.0292) but not when compared with tTA mice (*p* = 0.2115). Interestingly, a significantly higher level of CHOP protein was also observed in 3-month-old tTA mice compared with age-matched WT (*p* < 0.0001) or rTg4510 mice (*p* = 0.0035). The observed late-stage increase in CHOP protein occurs without accompanying changes in other UPR markers, suggesting that it may not be related to ER stress. Thus, we found no compelling evidence for UPR activation across key stages of the time course that encompasses the progressive pathologies expressed in the rTg4510 model of tauopathy.

### Primary neurons are susceptible to the UPR

**Figure 4.**
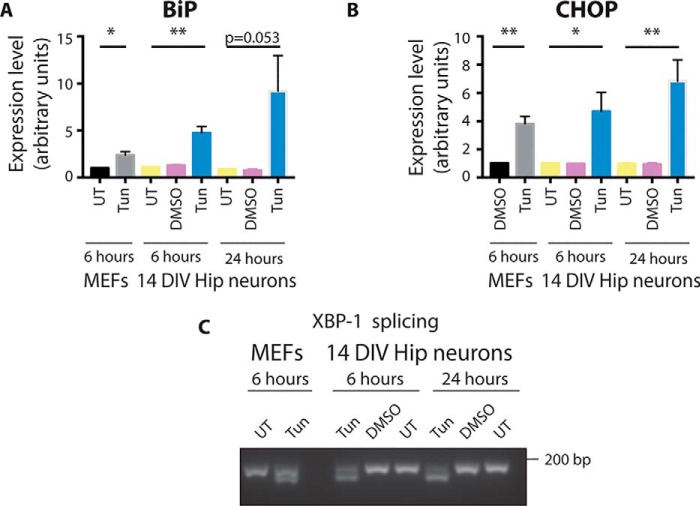
**Tunicamycin can induce the UPR in primary neurons.** *A*, expression level of BiP mRNA in primary neurons treated with tunicamycin (*Tun*) or DMSO or left untreated (*UT*) for 6 and 24 h. MEFs treated with tunicamycin were run alongside as a positive control. The expression level was normalized to the eIF4a expression level. *Error bars* are S.E. *, *p* < 0.05; **, *p* < 0.01. *n* = 3. *DIV*, days *in vitro. B*, expression level of CHOP mRNA in primary neurons treated with tunicamycin or DMSO or left untreated for 6 and 24 h. MEFs treated with tunicamycin were run alongside as a positive control. The expression level was normalized to the eIF4a expression level. *Error bars* are S.E. *, *p* < 0.05; **, *p* < 0.01. *n* = 3. *C*, RNA was extracted from primary neurons treated with tunicamycin or DMSO or left untreated for 6 and 24 h and subjected to PCR with primers detecting spliced and unspliced forms of XBP-1. The products were run on an agarose gel. MEFs treated with tunicamycin were run alongside as a positive control.

The lack of UPR activation in the model of progressive tauopathy may suggest that hippocampal neurons are not prone to undergoing UPR activation. To test that hypothesis, primary hippocampal neurons were treated with tunicamycin for 6 or 24 h. MEF cells, which are able to activate the UPR, were treated with tunicamycin as a positive control. The increase in BiP expression in hippocampal neurons was seen after 6-h treatment, and it remained up-regulated at the 24-h time point (Fig. 4*A*). Similarly, the induction of CHOP mRNA level was already detected after 6 h of treatment (Fig. 4*B*).

To further confirm the ability of neurons to undergo the UPR, RNA was extracted from primary neurons treated with tunicamycin for 6 or 24 h, and the splicing of XBP-1 was examined. The IRE-1 branch of the UPR was already activated after 6 h of treatment; two bands corresponding to the unspliced and spliced form of XBP-1 were present under both 6- and 24-h treatment conditions (Fig. 4*C*). These results indicate that harsh treatment, such as applying a glycosylation inhibitor, leads to activation of the UPR in primary neurons, showing the susceptibility of neurons to this response.

### Expressing tauP301L in primary neurons does not result in UPR activation

**Figure 5.**
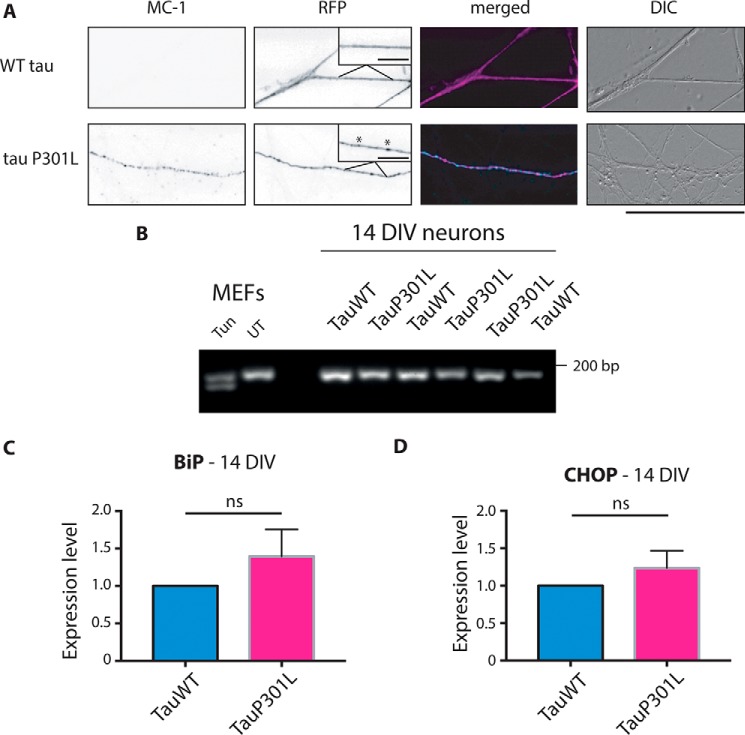
**UPR in primary neurons expressing tauP301L.** *A*, images comparing the axons of the neurons expressing tau^P301L^-RFP and tau^WT^-RFP cultured for 14 days *in vitro*. MC-1 staining in tau^P301L^-transfected cells suggests the presence of conformationally changed tau. The co-localization of MC-1 staining (*cyan*) and tau^P301L^-RFP (*magenta*) confirms that staining is present only in mutant tau-transfected cells and not in the surrounding untransfected axons. The *insets* show higher magnifications of the axon, with *asterisks* indicating the aggregates. *Scale bar* = 50 μm. *Scale bars* (*insets*) = 10 μm. *B*, primary neurons electroporated with tau^P301L^ and tau^WT^ were subjected to PCR with XBP-1 primers, and the products were resolved on a 2.5% agarose gel. MEFs treated with tunicamycin (*Tun*) were run alongside as a positive control. The electroporation efficiency was consistently at 70–80%. *n* = 3. *DIV*, days *in vitro*; *UT*, untreated. *C*, qPCR results showing the expression level of BiP transcript primary neurons electroporated with tau^P301L^ and tau^WT^. The expression level was normalized to the expression level of human tau. *n* = 3. *ns*, not significant. *D*, qPCR results showing the expression level of CHOP transcript primary neurons electroporated with tau^P301L^ and tau^WT^. The expression level was normalized to the expression level of human tau. *Error bars* are S.E. *n* = 3. *DIC*, differential interference contrast.

Having found that neurons are able to induce the UPR, we next examined whether misfolding of tau in cultured hippocampal neurons can drive UPR activation. This approach allowed us to isolate the effects triggered by pathogenic tau^P301L^ and eliminate confounds resulting from tTA expression, which are particularly pronounced at the early time points we investigated in the Tg4510 model. Expression of RFP-tau^P301L^ in hippocampal neurons resulted in the presence of red fluorescent aggregates along the axon that are not observed in RFP-tau^WT^-expressing neurons (Fig. 5*A*). Immunostaining with the conformation-specific antibody MC-1 (17) confirmed that these aggregates report misfolded tau. Importantly, MC-1 staining was observed only in tau^P301L^-transfected cells, whereas no immunoreactivity could be detected for tau^WT^-transfected neurons (Fig. 5*A*). Thus, this approach can be used to verify whether misfolded tau and the associated dysfunctional proteostasis drive UPR activation.

At day *in vitro* 14, no XBP-1 splicing was observed in tau^P301L^- or tau^WT^-electroporated cells (Fig. 5*B*), whereas both bands were present in tunicamycin-treated MEFs. To examine the expression of other UPR markers, qPCR experiments for BiP and CHOP were performed. Neither of the genes was significantly different between neurons electroporated with tau^P301L^ and tau^WT^ (*t* test, *p* = 0.3360 and *p* = 0.3698, respectively) (Fig. 5, *C* and *D*), regardless of whether the expression was normalized to a housekeeping gene or to the independently quantified expression of exogenous human tau. These results suggest that the presence of mutated and misfolded tau does not lead to induction of the UPR.

## Discussion

Here we tested whether the UPR is activated by the presence of misfolded tau. We tested both a mouse model of tauopathy as well as primary hippocampal neurons *in vitro*. This gave us traction to investigate the phenomenon in two distinct but complimentary systems.

The UPR is a homeostatic mechanism that can become dysregulated and has been discussed as a significant driver of pathology across many neurodegenerative diseases. This concept has been reinforced by preclinical studies showing that targeting arms of the UPR with inhibitors has therapeutic potential (18–20). Tau is one of the proteins whose aggregation is found in neurodegenerative diseases, and it has been reported that the presence of misfolded tau can induce the UPR (18, 20). However, it raises the important question of how the cytosolic aggregates of tau protein, which do not enter the ER lumen, can induce a response designed to counteract protein misfolding within the ER lumen. As UPR activation has been demonstrated previously in the rTg4510 model of tauopathy (18, 20, 21), we decided to use this model to explore how cytosolic misfolding triggers an ER luminal response.

We tested four commonly used markers of the UPR (XBP-1, p-eIF2α, BiP, and CHOP) and found no indication that the UPR is activated in rTg4510 mice. A significant change of rTg4510 compared with WT mice was observed for BiP protein in 9-month-old mice, but an increase was also observed in mice expressing the activator transgene tTA alone, suggesting that the change is not driven by mutant tau expression. A tTA-induced effect can also be seen for CHOP protein levels in 3-month-old mice, which display significantly increased levels compared with WT and rTg4510 mice. This suggests that the neurons that develop in the presence of the tTA transgene are not indifferent to its expression. Indeed, tTA expression in mice is associated with a range of changes, including a reduction in the number of dentate granule neurons and alterations in gene expression (12, 22).

The rTg4510 model has been used previously to investigate UPR activation. Radford *et al.* (18) observed no splicing of XBP-1, suggesting lack of activation of the IRE-1 pathway, which is consistent with our data. However, increased levels of other UPR markers, including p-eIF2α and p-PERK, were observed in other studies where WT mice were compared with rTg4510 mice (18, 20). We could not see a difference in the level of phosphorylated eIF2α in our samples using an antibody directed against the same phospho-epitope (Ser-51) as the one in the studies of Radford *et al.* (18) and Halliday *et al.* (20). We were also unable to detect p-PERK in rTg4510 samples (Fig. S1) and found no evidence of activation of the PERK branch of the UPR in response to tau pathology *in vivo*.

To further investigate UPR activation as a response to tau misfolding, we used an *in vitro* approach where we transfected primary neurons with tau^P301L^ or tau^WT^. In this system, we observed tau misfolding upon mutant tau expression, suggesting that it can be used to model tau pathology. Exogenously expressed RFP-tau appeared to be cytosolic or microtubule-associated in nature, with no reticulate pattern observed (data not shown). The culture approach allowed us to isolate the effects of tau^P301L^ expression from those of tTA expression. Comparing tau^WT^- and tau^P301L^-electroporated cells revealed no difference in the expression of UPR markers. This further suggests that the up-regulation of UPR components observed in rTg4510 mice is due to the presence of the tTA component as opposed to the accumulation of mutated tau.

Similar observations concerning the lack of UPR activation by mutant tau were made when using HEK293 cells expressing tau^P301L^, with tau expression controlled by the tetracycline-regulated T-Rex expression system. Neither splicing of XBP-1 nor induction of protein markers of the UPR were observed in this system upon the overexpression of recombinant tau (23).

Our results highlight the need to carefully control any data obtained from transgenic models based on the tetracycline transactivator system. The tTA system contains DNA- and tetracycline-binding domains of the *Escherichia coli* tetracycline repressor and the transcriptional activation domain of herpes simplex virion protein 16 (24). This latter component likely interacts with endogenous transcription factors, leading to the changes observed in tTA-expressing mice. For example, it has been shown that the presence of the tTA transgene leads to alterations in gene expression in the myocardium. Among the changing genes were those involved in metabolism and those encoding different chaperones (25). Large differences in gene expression between brains from WT and tTA mice have also been reported (22).

It has been questioned previously whether UPR activation occurs in neurodegenerative models. Hashimoto *et al.* (26) detected no UPR induction in amyloid precursor protein knockin mice, single APP-overexpressing mice, and a distinct model of tauopathy: P301S Tau-Tg mice. They suggested that the UPR activation observed for different transgenic models may be an artifact resulting from the expression of membrane proteins that could generate nonspecific ER stress. The lack of UPR activation in the tauopathy model could be explained by the cytosolic localization of tau protein, with no indication of tau entering the ER lumen (26).

On the other hand, other studies have reported neuroprotective effects in rTg4510 mice following treatment with GSK2606414. This drug was used on the bases that it acts as an inhibitor of PERK and that its inhibition would execute its effect by interfering with the UPR. The treatment decreased the levels of tau phosphorylation, reduced brain atrophy, and prevented behavioral scores of dysfunction (18). Of note, the inhibitor used in the cited study is not PERK-selective. It has been shown that it can potently inhibit receptor-interacting serine/threonine protein kinase 1 (27). Therefore, the impressive improvement of rTg4510 mice in response to GSK2606414 treatment may be a result of inhibiting necroptosis rather than the UPR.

Conflicting results have been reported for human cases of AD. BiP levels have been reported to be increased, decreased, or not changed between AD and control brains by different research groups (27, 28, 29). Hoozemans *et al.* (28) reported increased BiP expression using Western blotting and immunohistochemistry, whereas Sato *et al.* (29) did not detect an increase between control and familial or sporadic cases of AD. Katayama *et al.* (30) observed decreased levels of BiP in both sporadic and familial AD cases compared with nondemented controls. Lee *et al.* (31) reported an increase in XBP-1 splicing in AD cases; however, they observed the spliced form of the gene in control brains as well, suggesting that the splicing event is not a disease-specific feature. We have seen similar variation between AD and an age-matched nondemented cohort,^3^ highlighting the intrinsic difficulty of quantifying such stress pathways in pathological tissue (26). In conclusion, although there is good evidence for UPR activation in some neurodegenerative diseases, its causative role across neurodegeneration is less clear, particularly in view of the neurodegeneration-independent activation of the UPR observed in some mouse models.

## Experimental procedures

### Materials

The primary antibodies used in this study were anti-actin (Sigma, AC-40), anti-BiP (Cell Signaling Technology, 3177), anti-CHOP (Cell Signaling Technology, 2896), anti-p-eIF2α (Cell Signaling Technology, 3398), anti-eIF2α (Cell Signaling Technology, 9722), anti-tau (Dako, A0024), anti-GFAP (Dako, Z0334), anti-NeuN (Cell Signaling Technology, 12943), anti-GAPDH (Abcam, ab8245), and MC-1 and PHF-1 (gifts from Prof. Peter Davies) (17). Secondary antibodies were conjugated to Alexa Fluor fluorophores (Invitrogen) or IRDyes (LI-COR Biosciences). The plasmids pRK5-EGFP-TauP301L and pRK5-EGFP-Tau were gifts from Karen Ashe (Addgene plasmids 46908 and 46904, respectively) (32), and GFP in the constructs was exchanged for RFP.

Primers were obtained from Eurofins Genomics: BiP, 5-ATTGGAGGTGGGCAAACCAA-3 (forward) and 5-TCGCTGGGCATCATTGAAGT-3 (reverse); CHOP, 5-TCCCCAGGAAACGAAGAGGAAG-3 (forward) and 5-TCATGCGTTGCTTCCCAGGC-3 (reverse); XBP-1, 5-GGAGTGGAGTAAGGCTGGTG-3 (forward) and 5-GTCCAGAATGCCCAAAAGGATA-3 (reverse). Primers for the reference gene eIF4a were purchased from Primer Design.

### Mice

rTg4510 and age-matched WT and tTA mice were provided by Lundbeck (Denmark). Mice were euthanized by cervical dislocation, and the brains were quickly removed and portioned. One hemisphere was immersion-fixed in 4% paraformaldehyde for 36 h and processed for histology. The other hemisphere was snap-frozen on dry ice and stored at −80 °C until use. All rTg4510 experiments were performed in accordance with European Communities Council Directive 86/609, the directives of the Danish National Committee on Animal Research Ethics, and Danish legislation on experimental animals. For tissue collection, C57Bl/6 WT mice were sacrificed in accordance with the Animals (Scientific Procedures) Act 1986 as approved by the United Kingdom Home Office.

### Cell culture

Primary neurons were prepared in Dulbecco's PBS (Life Technologies) from hippocampi of E15–E18 C57Bl/6 mice. Neurons were dissociated as described previously (33). The number of cells was determined, and the cells were either plated or subjected to electroporation.

Electroporation was performed using the Mouse Neuron Nucleofector® Kit (Lonza) according to the manufacturer's instructions. After the electroporation procedure, neurons were incubated in recovery medium containing complete Neurobasal medium with 2% B27 supplement and 0.5 mm Glutamax with an additional 10% FBS at 37**°**C for 45 min. The electroporated cells were resuspended in Neurobasal medium and plated. The electroporation efficiency was consistently around 70–80%.

MEFs were isolated from E15 C57BI/6 mice and maintained in DMEM with 10% FBS. For tunicamycin treatment, the cells were treated with tunicamycin (2 μg/ml, Abcam) or 0.1% DMSO (vehicle treatment) prepared in medium. The cells were incubated at 37 °C for 6 or 24 h and then harvested.

### Immunocytochemistry

Cells were transfected on day *in vitro* 1 with Lipofectamine 2000 as described previously (30). The lipoplex mixture was added to the cells and incubated at 37**°**C for 40 min. Subsequently, the lipoplex-containing medium was removed from the wells and replaced with conditioned medium, and the cells were returned to the 37 °C.

On day *in vitro* 14, the cells were fixed and stained with MC-1 antibody (1:300). Alexa Fluor 488 was used as a secondary antibody. Hoechst stain was used to stain the nuclei.

The cells were imaged using a ×60/1.42 NA Oil Plan Apo objective on a DeltaVision Elite system (GE Life Sciences) with an SSI 7-band light-emitting diode for illumination and a monochrome sCMOS camera using SoftWoRks software (version 6). 4′,6-diamidino-2-phenylindole/FITC/TRITC channels were used. The images were analyzed using the Fiji processing package (34).

### Immunohistochemistry

Mice were perfused using 0.9% saline and then 4% paraformaldehyde. The mouse brains were fixed in 4% paraformaldehyde for 48 h and then in 30% sucrose for 48 h. A microtome was used to cut 30-μm-thick coronal sections. The sections were permeabilized in blocking solution (Tris-buffered saline containing 10% normal goat serum (Invitrogen), 2% BSA and 0.4% Triton X-100, both from Fisher Scientific) at room temperature for 2 h. Next, the sections were incubated in blocking solution containing NeuN antibody (1:500) overnight at 4**°**C. Incubation with primary antibody was followed by incubation with blocking solution containing Alexa Fluor 555 secondary antibody (Life Technologies, 1:500) for 2 h at room temperature. Before mounting the brain sections onto glass, sections were incubated in PBS containing Hoechst stain (1:10,000, Invitrogen) for 10 min.

Overview images were acquired using a Nikon Eclipse E800 microscope using ×4/0.2 NA Plan Apo objective with an Optimus sCMOS camera. 4′,6-diamidino-2-phenylindole/TRITC channels were used to take images. Images of the CA1 region were acquired using a Leica SP8 scanning confocal microscope using ×40/1.30 NA HC PI Apo CS2 oil immersion objective with a PCO Edge 5.5 sCMOS camera. Wave solid-state lasers at 405 and 561 nm were used for illumination. The images were analyzed using Fiji software (34).

### Tissue homogenization

The hippocampi and cortices from WT, tTA, and rTg4510 mice were homogenized in sterile PBS containing cOmplete Mini EDTA-free Protease Inhibitor Mixture tablets (Roche), sodium fluoride (10 mm, Fisher Scientific), and sodium orthovanadate (2 mm). The samples were homogenized using a Kontes pellet pestle motor and plastic pellet pestles. The samples homogenized in PBS were then used for RNA or protein extraction (35).

### qPCR

RNA was isolated from the hippocampi of WT, tTA, and rTg4510 mice using the TRIzol method. The RNA was then purified using the RNeasy kit (Qiagen). RNA from the cells was isolated and purified using the RNeasy kit. RNA extracted from hippocampi and cells was reverse-transcribed using the Precision nanoScript 2 reverse transcription kit (Primer Design) according to the manufacturer's instructions.

qPCR was performed using Precision PLUS Mastermix (Primer Design) according to the manufacturer's instructions. Primers specific for detection of mouse BiP and CHOP were used to detect the respective mRNA levels. qPCR was performed using the StepOnePlus real-time PCR instrument (Applied Biosystems) using the following conditions: hot start 95**°**C for 2 min, 40 cycles of denaturation at 95 °C for 10 s, and data collection at 60 °C for 1 min.

All reactions were performed with two technical replicates. The expression level was normalized to the level of the reference gene eIF4a.

### XBP-1 splicing

End-point PCR was performed using RedTaq ReadyMix PCR Reaction Mix (Sigma-Aldrich) and primers detecting both spliced and unspliced forms of XBP-1. PCR was performed using GeneAmp PCR System 9700 (Applied Biosystems) and the following conditions: hot start at 94 °C for 2 min and 40 cycles of denaturation at 94 °C for 40 s, annealing at 60 °C for 30 s, and extension at 72 °C for 1 min. Then the final extension was performed at 72 °C for 10 min. The products of the PCR reaction were separated on a 2.5% agarose gel.

### Western blotting

The proteins were extracted from PBS-homogenized hippocampi and cortices using 2× homogenization buffer containing HEPES-NaOH (40 mm (pH 7.4), Fisher Scientific), NaCl (250 mm, Sigma-Aldrich), SDS (4%), cOmplete Mini EDTS-free Protease Inhibitor Mixture tablets (Roche), sodium fluoride (10 mm), and sodium orthovanadate (2 mm). The samples were mixed with 5× sample buffer containing Tris (312.5 mm (pH 6.8)), SDS (10%), glycerol (50%), DTT (25 mm), and bromphenol blue dye (0.005%). The cells were lysed in 2× sample buffer containing Tris-HCl (125 mm (pH 6.8)), SDS (4%), glycerol (20%), DTT (10 mm), and bromphenol blue dye (0.002%),

The samples were boiled for 10 min at 95**°**C, spun, and then separated by SDS-PAGE and transferred to nitrocellulose membranes. The membranes were blocked in 2.5% BSA and TBS-T (Tris-buffered saline supplemented with 0.1% Tween20) for 1 h and then incubated at 4 °C overnight with the primary antibody. The membrane was then incubated with the secondary antibody at room temperature for 1 h. Images were captured using the Odyssey IR Imaging System (LI-COR Biosciences). Image Studio Scanner software was used to obtain the image, and Image Studio Lite software was used to quantify the intensities of the bands.

## Author contributions

A. P. P., V. O., and K. D. formal analysis; A. P. P. investigation; A. P. P. visualization; A. P. P. and A. A. A. methodology; A. P. P., V. O., and K. D. writing-original draft; A. P. P., A. A. A., V. O., and K. D. writing-review and editing; A. A. A. resources; V. O. and K. D. conceptualization; V. O. and K. D. supervision; V. O. and K. D. funding acquisition.

## Supplementary Material

Supporting Information

## Footnotes

3 A. P. Pitera, A. A. Asuni, V. O'Connor, and K. Deinhardt, unpublished observations.

## Footnotes

2 The abbreviations used are:

UPR: unfolded protein response
ER: endoplasmic reticulum
AD: Alzheimer's disease
PERK: protein kinase R–like endoplasmic reticulum kinase
tTA: tetracycline-controlled transactivator protein
cDNA: complementary DNA
ANOVA: analysis of variance
MEF: mouse embryonic fibroblast
qPCR: quantitative PCR
E15: embryonic day 15
NA: numerical aperture
TRITC: tetramethylrhodamine isothiocyanate
BiP: Binding immunoglobulin protein
GFAP: glial fibrillary acidic protein
CHOP: C/EBP Homologous Protein
RFP: red fluorescent protein
FITC: fluorescein isothiocyanate.
