# Supplementary material for: Pathogenic tau does not drive activation of the unfolded protein response
Source: J Biol Chem. 2019 May 3;294(25):9679–88. doi: 10.1074/jbc.RA119.008263 (PMC6597832; doi:10.1074/jbc.RA119.008263)
Supplement: Supporting Information [file supp_294_25_9679__index.html]

Pathogenic tau does not drive activation of the unfolded protein response — UPR is not induced in tauopathy — Pathogenic tau does not drive activation of the unfolded protein response — The UPR is not induced in tauopathy — Supporting Information 

# Pathogenic tau does not drive activation of the unfolded protein response

## Supporting Information

- Figure S1 - p-PERK is undetectable in rTg4510 animals.
