# Supplementary material for: Pathogenic tau does not drive activation of the unfolded protein response
Source: J Biol Chem. 2019 May 3;294(25):9679–88. doi: 10.1074/jbc.RA119.008263 (PMC6597832; doi:10.1074/jbc.RA119.008263)
Supplement: Supporting Information [file supp_RA119.008263_144075_2_supp_323655_pqty3k.docx]

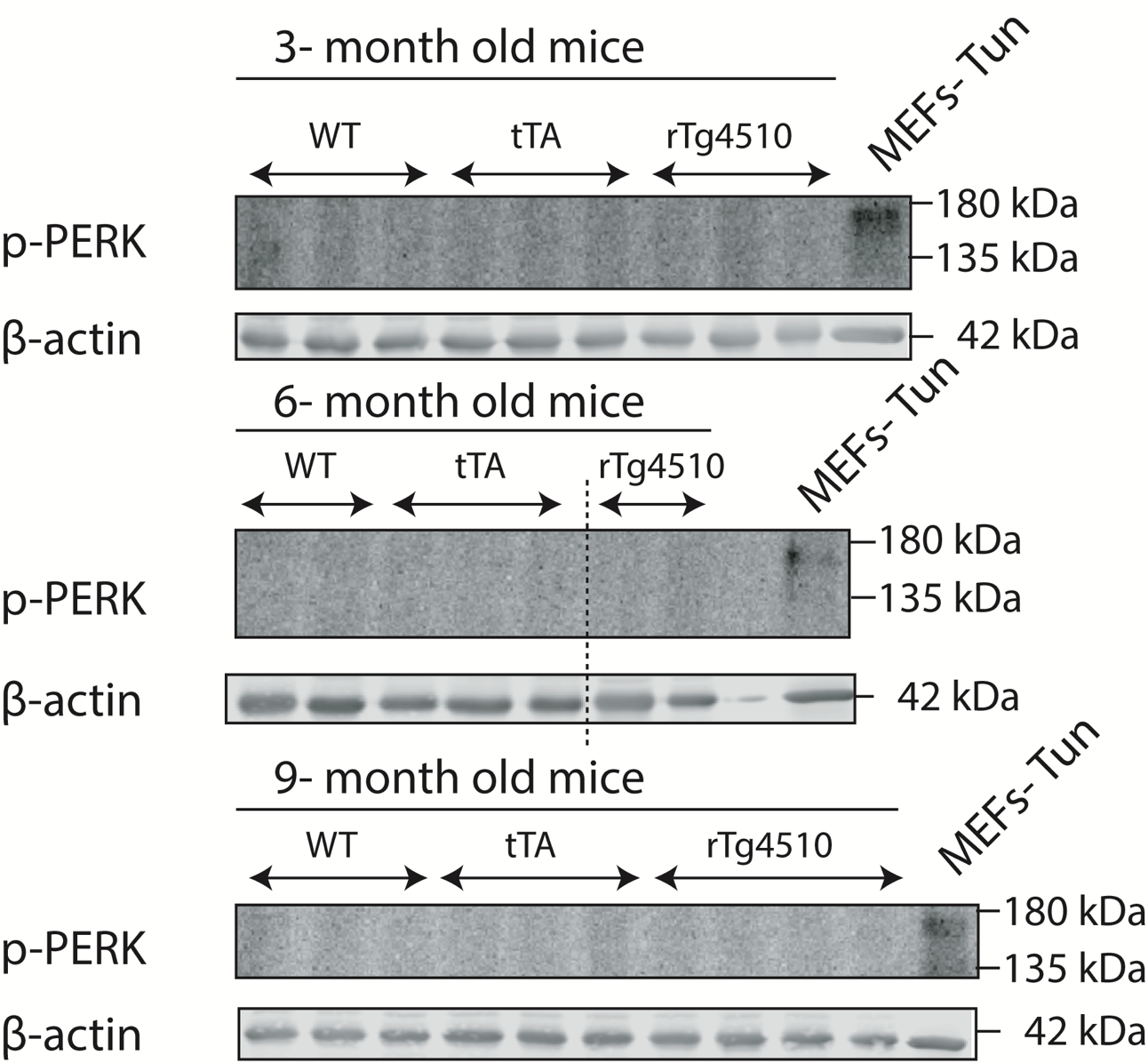


**Figure S1: p-PERK is undetectable in the rTg4510 animals.** Western blot showing the level of p-PERK in 3, 6 and 9-month old WT, tTA and rTg4510 mice. 40 µg of protein was loaded per sample. The blots from figure 2D were reprobed with p-PERK antibodies; the β-actin signal (also see figure 2D) illustrates equivalent protein loading. Each lane corresponds to a separate animal. Dashed line indicates splicing of the scan. Phospho-PERK Thr980 (16F8) antibody from Cell Signaling (3179) detected the band of correct size in the tunicamycin-treated MEF cells but no bands were observed for murine brain samples using fluorescent-based (LiCOR, not shown) and enhanced chemiluminescence-based (shown above) methods of detection.
